# Costs of implementing integrated community case management (iCCM) in six African countries: implications for sustainability

**DOI:** 10.7189/jogh.07.010403

**Published:** 2017-06

**Authors:** Emmanuelle Daviaud, Donnela Besada, Natalie Leon, Sarah Rohde, David Sanders, Nicholas Oliphant, Tanya Doherty

**Affiliations:** 1 Health Systems Research Unit, South African Medical Research Council, Cape Town, South Africa; 2 School of Public Health, University of the Western Cape, Cape Town, South Africa; 3 UNICEF, New York, NY, USA

## Abstract

**Background:**

Sub–Saharan Africa still reports the highest rates of under–five mortality. Low cost, high impact interventions exist, however poor access remains a challenge. Integrated community case management (iCCM) was introduced to improve access to essential services for children 2–59 months through diagnosis, treatment and referral services by community health workers for malaria, pneumonia and diarrhea. This paper presents the results of an economic analysis of iCCM implementation in regions supported by UNICEF in six countries and assesses country–level scale–up implications. The paper focuses on costs to provider (health system and donors) to inform planning and budgeting, and does not cover cost–effectiveness.

**Methods:**

The analysis combines annualised set–up costs and 1 year implementation costs to calculate incremental economic and financial costs per treatment from a provider perspective. Affordability is assessed by calculating the per capita financial cost of the program as a percentage of the public health expenditure per capita. Time and financial implications of a 30% increase in utilization were modeled. Country scale–up is modeled for all children under 5 in rural areas.

**Results:**

Utilization of iCCM services varied from 0.05 treatment/y/under–five in Ethiopia to over 1 in Niger. There were between 10 and 603 treatments/community health worker (CHW)/y. Consultation cost represented between 93% and 22% of economic costs per treatment influenced by the level of utilization. Weighted economic cost per treatment ranged from US$ 13 (2015 USD) in Ghana to US$ 2 in Malawi. CHWs spent from 1 to 9 hours a week on iCCM. A 30% increase in utilization would add up to 2 hours a week, but reduce cost per treatment (by 20% in countries with low utilization). Country scale up would amount to under US$ 0.8 per capita total population (US$ 0.06–US$0.74), between 0.5% and 2% of public health expenditure per capita but 8% in Niger.

**Conclusions:**

iCCM addresses unmet needs and impacts on under 5 mortality. An economic cost of under US$ 1/capita/y represents a sound investment. Utilization remains low however, and strategies must be developed as a priority to improve demand. Continued donor support is required to sustain iCCM services and strengthen its integration within national health systems.

Although under–five mortality reduced globally from 91 deaths/1000 live births in 1990 to 43/1000 (53% reduction) in 2015, sub–Saharan Africa still has the highest under–five mortality rate, despite a 54% decline from 180/1000 in 1990 to 83/1000 in 2015 [1,2]. The major causes of these deaths are largely preventable – neonatal disorders, diarrhea, pneumonia and malaria – for which low cost, high impact interventions and effective delivery strategies exist [1]. However, access to health facilities is poor, especially for families in rural and hard to reach areas [2].

Following the development of community health worker (CHW) programmes in the 1970s, and their decline at the end of the 80s, there is renewed interest in CHWs to improve access to services particularly in the context of task–shifting [3], with increasing evidence of their effectiveness in providing preventive and curative services [4–6]. In 2012, WHO and UNICEF issued a joint statement on integrated community case management (iCCM) as an equity–focused strategy to improve access to essential treatment services for children [7], with integrated diagnosis, treatment and referral services for malaria, suspected pneumonia and diarrhea among children aged 2–59 months (hereafter referred to as under–five) by trained and equipped CHWs. iCCM may also cover treatment of severe acute malnutrition and newborn illness [8].

Following the UNICEF endorsement, uptake of the strategy by national governments was rapid, from 7 countries in sub–Saharan Africa in 2005 to 28 by 2013, spearheaded by donor–driven initiatives providing a major share of funding [8]. Understanding the resources required to implement and scale up iCCM is critical for both governments and funders to assess value for money and affordability. This paper presents the results of an economic analysis of iCCM implementation in six sub–Saharan African countries, identifying factors which affect cost of treatment and possible areas of greater efficiency to support scale–up and affordability. This paper focuses specifically on cost to government and donors, the provider perspective. It does not cover costs to households, nor does it assess cost–effectiveness.

This analysis was part of a multi–country evaluation of the Catalytic Initiative/ Integrated Health Systems Strengthening program (CI/IHSS) in 2012–2013 [9]. These programmes were established by UNICEF with joint funding from the Department of Foreign Affairs, Trade and Development, Canada (DFATD) [10]. Ethiopia, Ghana, Malawi, Mali, Mozambique and Niger were selected for support.

CI/IHSS had a strong health systems strengthening focus (training, provision of drugs and supplies, support for supervision and development of monitoring and evaluation systems) [11]. Initially supporting mainly preventive interventions, it shifted focus to training and equipping CHWs to deliver iCCM services. This study focuses on those regions within countries supported by UNICEF, although other donors were supporting iCCM in additional regions.

## METHODS

We visited each country for approximately 10 days and interviewed Ministry of Health officials, partners, supervisors, CHWs, users and other stakeholders.

### iCCM program description

Community–based care existed in Ethiopia, Malawi and Niger focusing on mother and child and environmental issues, staffed with paid CHWs supervised by health centers staff. iCCM was added to their tasks. Ghana, Mali and Mozambique created or revitalized their CHW cadre. In Ghana iCCM is provided by volunteers while in the other countries CHWs are paid. The program was based on home visits (Ghana and Mozambique), combined with work from health posts (Ethiopia, Malawi, Mali and Niger). **Table 1** presents additional country information.

**Table 1:** Contextual factors

Context	Ethiopia	Ghana	Mali	Malawi	Mozambique	Niger
GDP per capita 2013 in 2015 US$	504	1827	660	240	605	419
Public Health Expenditure per capita 2013 in 2015 US$	15	60	21	13	19	10
Under 5 mortality per 1000 live births, 2013	64	78	123	68	87	104
% population living in rural areas	81	47	62	84	68	82
Pre–existing CHW cadre	Yes	Yes	No	Yes	Yes	Yes
**CHW characteristics:**						
Gender	All female	50% female	43% female	28% female	30% female	33% female
Educational background required	Grade 8–10	Most illiterate	9^th^ grade	12^th^ grade	7^th^ grade	12^th^ grade
Duration of basic training (in years)	1 year	5 days	40 days	3 months	4 months	6 months
Program design elements:						
Duration of iCCM training	6	3	15	6	23	6
Population <5 per CHW	377	72	360	632	735	576
Based in community or health post	Health post	Community	Health post	Health post	Community	Health post
Full monthly salary (US$)	40	Volunteer	80	110	40	100
Part of civil service	Yes	No	Yes	Yes	No	No, but paid by state grant
**Program implementation:**						
iCCM trained CHWs	27 116	16 812	1847	1018	905	2560
CHW attrition rate	4%	8%	4%	3%	3%	7%
CHWs/Supervisor	8	30	4	10	25	3
Average iCCM treatments/y/CHW	20	10	134	546	99	603
Hours on iCCM per CHW/week	1.2	1.0	3.1	7.2	3.0	8.6
Treatments per capita under 5 in 2013 in CI districts	0.05	0.27	0.27	0.46	0.14	1.05
**Time frame:**						
Design & set up	2007–2010	2007–2010	2007–2011	2007–2008	2007–2010	2007–2008
Implementation	2010–2013	2007–2013	2011–2013	2008–2013	2010–2013	2007–2013
Implementation year costed	2012–2013	2012–2013	2012–2013	2012–2013	2012–2013	2012–2013
Months since at scale (iCCM trained CHWs >80%)	11	36	2	11	13	35

CI – Catalytic Initiative, CHW – community health worker, iCCM – integrated community case management

### Costs

This analyses combines a budget–holder/program (UNICEF) and health systems perspective. Health systems costs included government provider time, while program costs, included training, bicycles, kits and commodities, allowances for supervisors and review meetings and CHW stipends in Mozambique and Mali. Costing of consumables and supervision was based on clinical protocols and supervision schedules. We used actual data for number of CHWs, size of target population and number of treatments delivered.

Incremental financial and economic costs are calculated. Financial costs reflect additional expenditure incurred for the program by UNICEF. Economic costs covered all resources up to district supervision, including financial costs (although annualisation of capital is calculated differently) and opportunity costs (value of time spent on program). In Mali the stipend was fixed, independent of time spent. In Mozambique 70% of the stipend was costed reflecting the share of Malaria, Diarrhea and Pneumonia (MDP) treatments among under–fives since CHWs also treat adults. For Ghana volunteers, time value was based on a basic agricultural worker’s wage [12].

Budget holder costs were collected retrospectively from UNICEF country offices financial records for the CI/IHSS districts: bicycles/motorbikes, CHW kits and life span per type of equipment, training, salaries/stipend, supervisors allowances, CHW and supervisors attrition rate, malaria positivity rate and unit costs of drugs and rapid diagnostic tests (RDT). All drugs and tests were sourced from UNICEF, apart from malaria supplies in Mozambique, local cost of drugs were provided by UNICEF country offices.

Data were also obtained for the CI regions on the numbers of CHWs trained, frequency and duration of supervision visits, attrition rates, number of treatments per condition, and number of under–fives targeted, from UNICEF and CI/IHSS program documents.

### CHW time

CHW time on iCCM was calculated as follows: 1) Average visit duration was set at 30 minutes (based on a previous costing study in Malawi [13]). 2) Travel time was added when iCCM was provided at household level. 3) An additional 20% of visits was added to reflect visits without treatment (eg, Malaria negative tests) while requiring CHW time; drawing on the malaria positivity rate, the share of malaria treatments in case load, and adding a small fraction for other visits without treatment 4) CHW time on supervision or community meetings and visits to the clinic for refilling of kits. 5) The above enabled to calculate the average time per week per CHW. It was assumed that a CHW works an average of 46 weeks per year. Time on iCCM was also used in the sensitivity analysis to assess time impact and feasibility of a 30% increase in demand.

### Time line and analysis time horizon

iCCM costs are incurred in 3 overlapping phases. First is the design phase (formative research, design of the intervention, of the training and of materials). These, often substantial, one–off costs are not included because they will not be incurred again if iCCM is scaled–up. Second is the set–up phase (purchase of equipment, training) and the third is the implementation phase. Phases 2 and 3 are the focus of this study. Set–up data were collected for several years, and for 1 year for the implementation phase: the year 2012/13, the only year where iCCM was at scale in the 6 countries (**Table 1**).

### Analysis

Set–up costs were annualized, using a 3% discount rate for economic costs [14] and straight depreciation for financial costs. In the perspective of assessing cost of an on–going program, annualisation of equipment cost was based on the life span of each piece of equipment. Similarly, we annualised training costs, not as per length of intervention, but of the likely life span of training in an on–going program: initial training for CHWs and supervisors was allocated 10 life years when refresher training–mentorship took place, and 5 years in countries without refresher training. CHWs and supervisors attrition rates were applied. Incremental government and budget–holder economic costs are presented separately and combined (**Table 2**). Costs are presented in US dollars 2015, updating 2013 local currency with local inflation rate [12], then translated into US$, using the 2015 local US$ exchange rate [15].

**Table 2:** Economic costs per provider and per treatment (2015 US$)

UNICEF cost per CHW	Ethiopia	Ghana	Mali	Malawi	Mozambiquez	Niger
Training	17	16	75	12	96	47
Equipment	13	44	88	36	57	74
Salary/stipend	–	–	834	–	160	–
Management & supervision	104	29	111	7	206	36
Other Overheads 5%	7	4	55	3	26	8
% ICCM	100	100	70	100	70	100
*Sub–total Fixed Cost per CHW*	*134*	*89*	*776*	*55*	*363*	*156*
Supplies (Drugs/Tests) per CHW	18	10	247	749	79	1859
Budget holder cost per CHW	152	99	1023	804	442	2015
Government cost per CHW:						
Training	3	2	7	–	9	1
Equipment	–	–	–	–	–	–
Salary/Stipend	31	10	–	238	–	307
Management & Supervision	51	22	69	93	118	37
Other Overheads 5%	4	2	4	17	6	17
% ICCM	100	100	70	100	70	100
*Sub–total Fixed Cost per CHW*	*85*	*33*	*53*	*332*	*89*	*345*
Supplies (drugs/tests) per CHW:						
Government cost per CHW	85	33	53	332	89	345
**Combined costs per CHW:**						
Training	20	18	82	12	105	47
Equipment	13	44	88	36	57	74
Salary/stipend	31	10	834	238	160	307
Management & supervision	155	51	179	100	324	73
Other Overheads 5%	11	6	59	19	32	25
% ICCM	100	100	70	100	70	100
*Sub–total Fixed Cost per CHW*	*219*	*122*	*828*	*386*	*452*	*502*
Supplies (drugs/tests) per CHW	18	10	247	749	79	1859
Total cost per CHW	237	132	1075	1135	531	2360
**Combined cost per treatment:**						
Number of iCCM treatments/CHW/year	20	10	134	546	99	603
Consultation cost/treatment	11.5	12.6	6.5	0.7	4.8	0.9
Average consumable/treatment	0.9	0.9	1.8	1.4	0.8	3.1
Economic cost/treatment	12.4	13.5	8.3	2.1	5.6	4.0
Share consultation cost	89	90	77	7	83	9
**Share government cost**	**36**	**25**	**5**	**29**	**17**	**15**
**Share budget holder**	**64**	**75**	**95**	**71**	**83**	**85**

CHW – community health worker, iCCM – integrated community case management

Fixed costs per CHW, independent of the number of treatments: annualised set–up and 1 year implementation costs were combined to calculate annual fixed costs per CHW: CHW iCCM training, equipment, CHW salaries/stipends and allowances for meetings, supervision and management costs (iCCM training for CHWs’ direct supervisors and district/zonal supervisors, share of supervisors’ salary package, allowances for supervision and meetings, and bicycles or share of motorbike costs), and overheads of 5% of the annualised costs. The annualised fixed costs per CHW divided by the number of treatments per CHW in 2012/13 represent “the consultation cost”.

Variable costs included drugs and RDTs used. Malaria positivity rate was used to calculate the number of RDTs used per child testing positive: with a 40% positivity rate, if 100 children are treated for malaria, 250 children have been tested; with a positivity rate of 60%, 167 children need to be tested. The average cost of consumables by treatment is weighted by the relative share of malaria, diarrhea and pneumonia treatments.

**Figure 1:**
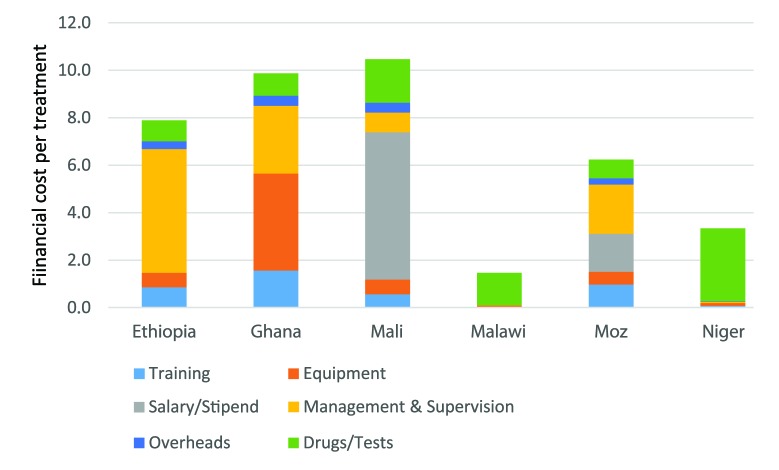
Financial cost per treatment (2015 US$).

Financial costs per treatment are presented in **Figure 1**, highlighting the share of consultation cost per treatment.

### Affordability

We used financial costs to calculate the budgetary implications of the program: cost per CHW and per capita total population in study areas, which we then expressed as a percentage of the country 2013 public health expenditure (PHE) per capita (government + donors), as a proxy for affordability of the program. Data on health expenditure and 2013 total and rural population were obtained from the World Bank databank [16]. The number of under–fives in 2013 was extracted from the UNICEF statistical Tables [17].

### Sensitivity analysis

We modeled the impact of a 30% increase in demand, keeping the ratio of children per CHW observed in the CI districts and assessed the implications for CHW time, consultation cost, cost per treatment and cost per capita. We then modeled the national program cost if the program was scaled up to all under–fives living in rural areas.

## RESULTS

### Country contexts

In Niger over 80% of the trained CHWs operated in 2013, in Mali the majority of CHWs were trained in 2011 and in Ethiopia, Ghana, Malawi and Mozambique the CHWs had only been functioning at that level for about a year (**Table 1**).

Annual number of iCCM treatments per CHW ranged from 10 in Ghana to 603 in Niger, influenced by the number of under–fives per CHW, from 72 in Ghana to 632 in Malawi.

Time on iCCM was estimated between 3 hours a week or less per CHW in Ethiopia, Ghana, Mali and Mozambique to around 1 full day in Malawi and Niger (**Table 1**).

### Fixed costs per CHW and consultation cost

Annualised financial fixed costs per CHW ranged from US$ 811 in Mali to US$ 55 in Malawi, lower costs reflecting the pre–existence of community–based care with CHWs and supervisors salaries already paid by the state. Financial cost per consultation ranged from US$ 0.1 in Malawi to US$ 9 in Ghana. Annualised economic fixed costs per CHW (**Table 2**) ranged from US$ 128 in Ghana to US$ 870 in Mali (**Figure 1**), with an economic cost per consultation between under US$ 1 in Malawi and Niger with high utilization, and over US$ 11 in Ethiopia and Ghana.

Training costs are higher in Mali and Mozambique, with longer training (15 and 23 days respectively) compared to 6 days in other countries. Annualised equipment costs: bicycles, if relevant, and kits (excluding consumables) ranged from US$ 88 in Mali to US$ 13 in Ethiopia. Management and supervision cost represented a significant share of CHW fixed cost, from US$ 324 in Mozambique to US$ 51 in Ghana, largely reflecting the ratio of CHWs per supervisor (**Table 1**) and supervisors’ daily allowance.

### Cost per treatment

Cost of consumables per condition varied, partly due to differences in protocols, with use of additional drugs (Paracetamol), or in Niger, using the more expensive drug ASAQ for malaria treatment. Positivity rates for malaria varied from 40% to 67%. Drug pricing systems also differed: in Mali, drugs distributed from clinics are 50% more expensive than at national level (distribution costs and revenue for the clinic) (**Table 3**).

**Table 3:** Cost of consumables per iCCM treatment (2015 US$)

	Ethiopia	Ghana	Mali	Malawi	Mozambique	Niger
**Share of treatments (%):**						
Malaria	30	49	59	54	40	54
Diarrhea	41	32	20	13	29	18
Pneumonia	29	19	21	33	31	27
Total	100	100	100	100	100	100
**Cost consumables per treatment (US$):**						
Malaria including rapid diagnostic test	1.74	1.56	2.73	2.32	1.51	4.84
Diarrhea	0.69	0.35	1.03	0.61	0.30	0.61
Pneumonia	0.26	0.39	0.09	0.13	0.33	1.24
**Economic cost per treatment including consultation (US$):**						
Malaria	13.3	14.1	9.2	3.0	6.3	5.7
Diarrhea	12.2	12.9	7.5	1.3	5.1	1.5
Pneumonia	11.8	12.9	6.6	0.8	5.1	2.1

Economic cost per malaria treatment ranges from US$ 3 in Malawi to US$ 14 in Ghana (financial cost US$ 2.4 to US$ 10.5 respectively), for diarrhea from US$ 1.3 to $12.9 (financial cost US$ 0.7 to US$ 9.3). User fees are implemented in two countries. In Mali patients pay US$ 0.2 per consultation and a weighted average of US$ 0.11 for drugs (excluding for malaria). In Ghana, patients contribute for drugs only, at a weighted average of US$ 0.34 per treatment (**Figure 1**).

### Affordability

Government paid from 5% of economic costs in Mali to under 20% in Mozambique and Niger, between 25% and 30% in Ghana and Malawi and 36% in Ethiopia (**Table 2**), while 100% of financial costs were paid by UNICEF. Financial costs range from US$ 0.06 per capita total population in Ethiopia to US$ 0.74 in Malawi and Niger, representing under 0.6% of the 2013 PHE per capita in Ethiopia, Ghana and Mozambique, but 1.8% in Malawi, 2.7% in Mali and 7.4% in Niger (**Table 4**), program cost per CHW ranges from US$ 101 in Ghana to US$ 2 047 in Niger (**Table 4**).

**Table 4:** iCCM impact on 2013 Public Health Expenditure (2015 US$)

	Ethiopia	Ghana	Mali	Malawi	Mozambique	Niger
Current utilization:						
Financial cost/CHW	158	101	1058	804	458	2047
Financial cost/capita total population	0.06	0.20	0.57	0.23	0.11	0.74
Share of 2013 Public Health Expenditure per capita (%)	0.4	0.3	2.7	1.8	0.6	7.4
Utilization +30%:						
Financial cost/CHW	163	104	1132	1029	482	2604
Financial cost/capita total population	0.07	0.20	0.61	0.29	0.11	0.94
Share of 2013 Public Health Expenditure per capita (%)	0.4	0.3	3	2.2	0.6	9.4
If scaled up to all rural areas:						
Share of 2013 Public Health Expenditure per capita (%)	0.4	0.2	1.8	1.8	0.4	7.7

### Implications of increased utilization

With 30% higher utilization, time on iCCM would increase by 10% to just over 1 hour a week per CHW in Ghana, but in Niger would be 28% higher to 11 hours a week. A 30% increase in utilization could be absorbed by the existing CHWs. In Ethiopia, Ghana, Mali, and Mozambique economic and financial costs per treatment would be about 20% lower. In Malawi and Niger, where consultation cost represents a small share of cost per treatment, the decrease is small (2 to 4%). Total costs of the program increase, however, due to additional consumables (**Table 5**). Financial costs of the program with higher utilization would remain under US$ 1 per capita.

**Table 5:** Impact on time and costs of increased utilization

	Ethiopia	Ghana	Mali	Malawi	Mozambique	Niger
Hours per week on iCCM:						
Current utilization	1.2	1.0	3.1	7.2	3.0	8.6
Utilization +30%	1.4	1.0	3.6	9.0	3.6	11.0
Economic cost per treatment:						
Current utilization	12.4	13.5	8.3	2.1	5.6	4.0
Utilization +30%	10.0	10.7	6.8	2.0	4.5	3.9
Decrease in cost per treatment	–19	–21	–18	–4	–20	–2
Increase in program cost	5	3	7	25	4	27
Additional financial cost/treatment:						
Current utilization	7.9	9.9	7.9	1.5	4.1	3.4
Utilization +30%	6.3	7.8	6.5	1.5	3.7	3.3
Decrease in cost per treatment (%)	–20	–21	–19	–3	–21	–2
Increase in program cost (%)	4	2	6	26	3	27

### Affordability of scale–up

If the program with 30% increased utilization was scaled–up to cover all the country under–fives living in rural areas, the cost per CHW would remain the same, iCCM would represent between 0.4% and 7.7% of the country PHE.

## DISCUSSION

Utilization of iCCM services in UNICEF supported districts varied from 0.05 MDP treatment per year per under–five in Ethiopia to over 1 in Niger. Low utilization does not appear to be mainly related to availability of services (supply side), but to demand, as documented in many countries [9]. In Ethiopia around 80% did not seek treatment for the 3 conditions, 60% in Ghana, 50% in Mozambique, and 40% in Niger, but much lower in Malawi at 13%. All countries reported low levels (10–15%) of care seeking from informal providers [9]. Demand does not seem linked to the size of the under 5 population per CHW, a proxy for availability of iCCM services: 377 children per CHW in Ethiopia and 576 in Niger. Medicine stock–outs were most marked in Malawi [9] but at 0,5 the number of treatments per child per year, was 10 times that of Ethiopia with low levels of stock outs. User fees contribute to low demand: user fees were reduced by two–thirds in an iCCM district in Mali, translating into demand more than doubling [18]. In Ghana patients registered for National Health Insurance received free treatment at clinics but paid for treatment by CHWs (half of the payment was used for CHW incentives), almost certainly deterring use. Utilization rates, through consultation cost, impacts directly on cost per treatment. They were the highest contributor to the efficiency, and likely effectiveness, of the programs, pointing to the necessity of proactive strategies to increase demand for existing programs before moving to scale–up.

Economic cost per consultation was US$ 12.6 in Ghana and US$ 0.7 in Malawi, representing 93% of cost per treatment in Ethiopia and Ghana but only 22% in Niger. Similar findings were reported in another multi–country assessment of iCCM costs [19].

Estimating CHWs time on the program is important to ensure that adding iCCM to CHWs’ workload does not squeeze out other existing activities and that increased utilization is manageable. Estimated time from 1 to 9 hours a week, represents a small portion of CHWs’ time whose main focus is mother and child health. A 30% increase in utilization would add under 1 to 2 hours a week, indicating that iCCM can be combined with other community–based activities: Across countries, communities requested that services be extended to older children, while CHWs indicated the difficulty of denying treatment to children over five in the same family.

iCCM training duration also impacted on costs: from 6 days in 3 countries to 23 in Mozambique. However it was only 3 days in Ghana for often illiterate volunteers without previous IMCI training, which may have contributed to low utilization.

Attrition rate has cost implications for training. It was under 4% for paid CHWs, except for Niger at 7% (clear career pathing to become a nurse may have contributed), and at 8% among Ghana volunteers. Similar differences between paid workers and volunteers were observed in another multi–country iCCM costing [19].

Management and supervision were a significant cost driver. Most countries however report recurrent challenges with consistent supervision including insufficient funding for supervisors travel and overwhelmed resource capacity; some of these could be addressed through mentorship at facility level, integrated supervision visits and use of simple supervision checklists [20].

The assumption that volunteers cost little, so many can be recruited, was contradicted by the Ghana example with the highest cost per treatment, due to fixed costs per volunteer and low utilization. With 2 CHWs per village, each covered an average of 72 children, compared to over 360 in the other countries. With one CHW per village, s/he would spend 1.2 hours a week on iCCM, average cost per treatment would be 47% lower, and incentive payments per CHW would double, emphasizing the need for more considered CHW deployment even in volunteer–based programs.

Scaling up the program to cover all rural areas would amount for all countries to under US$ 0.8 per capita total population, in most countries a small percentage of the PHE per capita: under 0.5% in Ethiopia, Ghana and Mozambique, under 2% in Malawi and Mali but 8% in Niger with the lowest PHE per capita. These countries are likely to remain dependent on foreign assistance to maintain and scale–up iCCM services which threatens their sustainability.

A systematic review of iCCM effectiveness reported a reduction in all–cause mortality in under–fives by up to 63% [21]. To sustain this, proactive support by governments is needed. Although iCCM has been recognized at policy level in these six countries, few have committed domestic resources. A survey of iCCM policy and implementation in sub–Saharan Africa reported that only 9 out of 33 countries had a budget line in government budget and CHW salaries were paid by government in only 5 countries [8]. In Mali there is uncertainty as to whether CHWs will be paid from user fees or funded by government/municipalities; the Mozambican government is reluctant to include CHWs as a new staff category of the public service (a pre–condition to allocate funds for their stipends). In Ghana CHWs are not included in the NHI.

In 2014 UNICEF, the Global Fund and the Reproductive, Maternal, Newborn and Child Health Trust Fund announced a unified plan to scale–up iCCM, with increased resources to expand in the near term [22].

iCCM costs need to be considered in light of expected savings from possibly reduced workload at clinics and from averting serious illness through early treatment. Families also experience time and cost savings with care closer to home. There is also increasing evidence of CHWs cost–effectiveness compared to increasing coverage of fixed health facilities staffed with nurses [23,24]. In Niger CHWs addressed unmet needs rather than replacing facility care seeking [25]. Costing of iCCM activities must also acknowledge that delivery of curative services strengthens the preventive role of CHWs [26].

### Limitations

This economic analysis has several limitations: first, in its scope: it focuses on resource implications of iCCM and affordability and does not include an analysis of value for money, a pre–requisite, but the programs were mostly too recent to assess effectiveness. Additionally, CHWs work was supported by multi–programs volunteers whose cost has not been included. Second, in its measurement methods: implementation costs were measured for 1 year, recent guidelines suggest that a wider window of implementation costs should be measured [27], however in this evaluation there was only 1 common year of implementation at scale. In addition, the normative approach used for calculating commodities and supervision costs has the benefit of estimating costs as per protocol, but does not reflect variations in actual implementation. CHW time on the program was based on the same assumptions of length of visit and meetings for all countries rather than on observation. However informal observation during country visits showed that our assumption of 30 minutes per visit is unlikely to be an underestimation. Another limitation is that the modeling of extending the program to all rural areas was made by extrapolating CI districts data and did not reflect other iCCM districts with potentially different cost profiles. Additionally affordability is based on per capita spending and does not include possible savings/costs to other levels of the health system as a consequence of the program in a way a Budget Impact Analysis would do [28].

A strength of this analysis is the separation of economic from financial costs to avoid double–counting in the calculation of budgetary implications. Second, rather than separating set–up costs from recurrent costs, annualised set–up and implementation costs are combined since over time equipment has to be replaced and training redone, and cannot be considered as one–off costs.

## CONCLUSIONS

By addressing at community level the three main causes of deaths of children aged 2–59 months, iCCM can service unmet needs and contribute to reductions in under 5 mortality. While the programs were mostly too recent to assess effectiveness, a financial cost in this study of under US$ 1 per capita per year, highlights that iCCM can represent a very sound investment. Once services have been built–up, strengthening demand must become the priority.

Aligning funding and support with national priorities, along with political will and commitment of governments is central for sustainability of iCCM and CHW platforms. Benefits from strategies such as iCCM is dependent on country context and economic outlook. Continued donor support is required for the foreseeable future and should have a concurrent focus on strengthening integration of iCCM as an essential platform of care within national health systems.
